# Resected pancreatic adenocarcinoma: An Asian institution's experience

**DOI:** 10.1002/cnr2.1393

**Published:** 2021-05-03

**Authors:** Kennedy Yao Yi Ng, Edwin Wei Xiang Chow, Bochao Jiang, Cindy Lim, Brian Kim Poh Goh, Ser Yee Lee, Jin Yao Teo, Damien Meng Yew Tan, Peng Chung Cheow, London Lucien Peng Jin Ooi, Pierce Kah Hoe Chow, Joycelyn Jie Xin Lee, Juinn Huar Kam, Ye Xin Koh, Prema Raj Jeyaraj, Ek Khoon Tan, Su Pin Choo, Chung Yip Chan, Alexander Yaw Fui Chung, David Tai

**Affiliations:** ^1^ Division of Medical Oncology National Cancer Centre Singapore Singapore; ^2^ Division of Clinical Trials and Epidemiological Sciences National Cancer Centre Singapore Singapore; ^3^ Department of Hepatopancreatobiliary and Transplantation Surgery Singapore General Hospital Singapore; ^4^ Division of Surgical Oncology National Cancer Centre Singapore Singapore; ^5^ Duke‐NUS Graduate Medical School Singapore; ^6^ Surgical Associates, National Cancer Centre Singapore Singapore; ^7^ Department of Gastroenterology and Hepatology Singapore General Hospital Singapore; ^8^ Curie Oncology, Graduate Medical School Singapore General Hospital Singapore

**Keywords:** pancreatic adenocarcinoma, prognostic factors, resected

## Abstract

**Background:**

Pancreatic adenocarcinoma (PDAC) is highly lethal. Surgery offers the only chance of cure, but 5‐year overall survival (OS) after surgical resection and adjuvant therapy remains dismal. Adjuvant trials were mostly conducted in the West enrolling fit patients. Applicability to a general population, especially Asia has not been described adequately.

**Aim:**

We aimed to evaluate the clinical outcomes, prognostic factors of survival, pattern, and timing of recurrence after curative resection in an Asian institution.

**Methods and Results:**

The clinicopathologic and survival outcomes of 165 PDAC patients who underwent curative resection between 1998 and 2013 were reviewed retrospectively. Median age at surgery was 62.0 years. 55.2% were male, and 73.3% had tumors involving the head of pancreas. The median OS of the entire cohort was 19.7 months. Median OS of patients who received adjuvant chemotherapy was 23.8 months. Negative predictors of survival include lymph node ratio (LNR) of >0.3 (HR = 3.36, *P* = .001), tumor site involving the body or tail of pancreas (HR = 1.59, *P* = .046), presence of perineural invasion (PNI) (HR = 2.36, *P* = .018) and poorly differentiated/undifferentiated tumor grade (HR = 1.86, *P* = .058). The median time to recurrence was 8.87 months, with 66.1% and 81.2% of patients developing recurrence at 12 months and 24 months respectively. The most common site of recurrence was the liver.

**Conclusion:**

The survival of Asian patients with resected PDAC who received adjuvant chemotherapy is comparable to reported randomized trials. Clinical characteristics seem similar to Western patients. Hence, geographical locations may not be a necessary stratification factor in RCTs. Conversely, lymph node ratio and status of PNI ought to be incorporated.

## 1 INTRODUCTION

Pancreatic adenocarcinoma (PDAC) is a highly lethal malignancy. It is the eighth leading cause of cancer‐related deaths in men and the ninth leading cause of cancer‐related deaths in women worldwide.^1^ PDAC often presents in advanced stages due to its aggressive biology and non‐specific symptoms.

The prognosis is poor even among patients with resectable disease, with a 5‐year survival of 10% to 30%.^2^ Current staging and prognostic tools rely on the American Joint Committee of Cancer (AJCC) TNM staging system eighth edition.^3^ Numerous other prognostic factors have been identified to better prognosticate patients with resected PDAC such as neutrophil/lymphocyte ratio,^4^ lymph node ratio,^5^ presence of lymphovascular invasion (LVI) or perineural invasion (PNI),^6^ and resection margin status.^7^, ^8^ The standard operation for tumors of the pancreatic head is a pancreaticoduodenectomy (Whipple procedure), whereas tumors of the body or tail can be resected using a distal pancreatectomy.^9^ These procedures are associated with high operative mortality and morbidity.^9^ Advancement in surgical technique and perioperative management of patients has led to a reduction in the morbidity and mortality associated with the above‐mentioned surgeries. Moreover, with the improvement of imaging technique and the employment of a multi‐disciplinary team approach, better selection of suitable patients for surgery could be done.^10^ Surgical outcomes at high‐volume centers have been shown to be superior compared to outcomes at low‐volume centers. In spite of that, many patients relapse at both local and distant sites after resection. Hence, adjuvant chemotherapy is crucial in the management of these patients as demonstrated in multiple randomized controlled trials (RCT).^11^, ^12^, ^13^, ^14^, ^15^, ^17^, ^18^, ^38^ Often, these trials stratify patients by geographical locations, resection margins, T‐stage and lymph node status. Adjuvant chemotherapy or chemoradiotherapy was conducted primarily in West enrolling fit patients with preserved organ functions and good performance status. Applicability to a general population especially in an Asian population has been inadequately described.

Pattern, timing, and predictors of recurrence after curative resection have been described primarily in Western populations.

We aimed to evaluate the clinical outcomes, prognostic factors of survival, pattern, and timing of recurrence after curative resection in an Asian institution.

We also compared the resected PDAC series from both Asian and Western populations.

## 2 METHODS

Patients who underwent resection with curative intent in our center between 1998 and 2013 were identified from a retrospective database. Patients eventually noted to have R2 resection or stage 4 disease were excluded. We collected clinicopathological and operative data of 165 patients. Follow‐up and data collection extended to December 2015.

Following surgery, all specimens underwent histopathological review, and features such as histology subtype, pathological AJCC stage and grade, resection margin status, tumor size, LVI and PNI. Resection margin involvement was defined according to the Royal College of Pathologists guidelines, with microscopic evidence of tumor within 1 mm of a resection margin (RM) being classified as R1.^19^ Laboratory parameters such as CA 19‐9 and carcinoembryonic antigen (CEA) were measured preoperatively and postoperatively (patients without tests done within 3 months before or after the surgery was excluded from the analysis). The development of a hypointense mass in the resection site was considered as evidence of local recurrence. Similarly, detection of a new hypointense nodule/mass in the liver, lung, or peritoneum was considered evidence of distant recurrence. No biopsies were performed in this series to confirm the diagnosis of recurrent cancer. If the CT findings were non‐specific, a follow‐up CT would be performed, and the date of recurrence will be taken as the date of the follow‐up CT that demonstrate enlargement of the nodule or mass. Our study was approved by the Centralized Institutional Review Board of our institution.

### 2.1 Statistical analysis

Continuous variables were summarized using median and range. Categorical variables were summarized using frequency and percentage. Overall survival (OS) was calculated as the time from surgery to death from all causes. Patients who were alive at last follow‐up were censored at date of last follow‐up. Median OS was estimated using the Kaplan‐Meier method. Differences in survival curves were tested using the log‐rank test. Univariable and multivariable analyses were performed using the Cox proportional hazards model. For multivariable analysis, variable selection was performed using a forward selection procedure. All variables, regardless of significance in univariable analysis, were entered as candidate variables in the forward selection procedure. Only variables with more than 10% missing data were excluded. The proportional hazards assumption was tested on the final multivariable model using a test based on Schoenfeld residuals. A *P*‐value of less than .05 was taken as statistically significant in the univariable analyses. For the forward selection procedure, a *P*‐value of less than .10 was used for addition of variables into the multivariable model. *P*‐values for Cox models were calculated using the likelihood ratio test. All analyses were performed in Stata 15.0 (StataCorp, College Station, Texas).

## 3 RESULTS

### 3.1 Study population characteristics

Our study population consisted of 165 patients with resected pancreatic ductal adenocarcinoma. Median age at surgery was 62.0 (41‐84) years. 55.2% were male and 44.8% were female. The ethnic proportion of our study population was 77.6% Chinese, 4.8% Malay, 4.2% Indian, and 12.7% of other races. The median follow‐up time was 15.5 months. Regarding grade of differentiation, 10.9% had well differentiated, 75.2% moderately differentiated, 12.1% poorly differentiated, and 0.6% undifferentiated histology. Majority (73.3%) of patients had tumors involving the head of pancreas. Whipple operation or pylorus‐preserving pancreaticoduodenectomy (PPPD) was the most common form of surgery (73.3%) followed by distal pancreatectomy in 22.4%, and total pancreatectomy in 2.4%. The institution's surgical outcomes and details were previously published.^20^, ^21^ Only 50.9% of patients who underwent curative resection eventually received adjuvant therapy. Of these, 55 (33.3%) received adjuvant chemoradiotherapy, 33 (20.0%) received only adjuvant chemotherapy and 1 (0.6%) received only adjuvant radiotherapy. No patients received neoadjuvant chemotherapy or chemoradiotherapy. All patients who received adjuvant chemotherapy received gemcitabine or 5‐fluorouracil (5‐FU)/oral capecitabine monotherapy. Patients receiving adjuvant chemoradiotherapy received either concurrent radiotherapy with radiosensitizing 5‐FU or gemcitabine followed by gemcitabine or 5‐FU monotherapy. Patient demographic and clinicopathologic characteristics of the cohort are detailed in Table 1.

**TABLE 1:** Patient demographics and clinical characteristics

Characteristic	Frequency	Percentage
Total number of patients	165	100
Age at surgery (years)		
Median (Range)	62 (41–84)	
Gender		
Male	91	55.2
Female	74	44.8
Race		
Chinese	128	77.6
Malay	8	4.8
Indian	7	4.2
Others	21	12.7
Unknown	1	0.6
Smoking status		
Never	86	52.1
Ex	30	18.2
Current	10	6.1
Unknown	39	23.6
Alcohol consumption		
Never	96	58.2
Ex	9	5.5
Current	19	11.5
Unknown	41	24.8
Charlson comorbidities index		
Median (Range)	3 (1–9)	
Symptoms		
Loss of weight	42	25.5
Loss of appetite	27	16.4
Fever	4	2.4
Abdominal pain	48	29.1
Abdominal distension	5	3.0
Diarrhea	4	2.4
Jaundice	84	50.9
Malaena	1	0.6
Tumor site		
Head involved	121	73.3
Head not involved	44	26.7
AJCC TNM stage		
IA	5	3.0
IB	16	9.7
IIA	45	27.3
IIB	90	54.5
III	9	5.5
T stage		
T1	4	2.4
T2	30	18.2
T3	123	74.5
T4	8	4.8
N stage		
N0	69	41.8
N1	95	57.6
NX	1	0.6
Histological grade		
Well differentiated	18	10.9
Moderately differentiated	124	75.2
Poorly differentiated	20	12.1
Undifferentiated	1	0.6
Not stated/not determined	2	1.2
Type of surgery		
Whipples operation or Pylori preserving pancreaticoduodenectomy (PPPD)	121	73.3
Pancreatectomy, distal or subtotal	37	22.4
Pancreatectomy, total	4	2.4
Pancreatectomy, NOS	3	1.8
Resection margins		
R0	80	48.5
R1	85	51.5
Perineural invasion		
No	14	8.5
Yes	135	81.8
Indeterminate	6	3.6
Unknown	10	6.1
Lymphovascular invasion		
No	80	48.5
Yes	62	37.6
Indeterminate	13	7.9
NA	10	6.1
Lymph node resected		
Median (Range)	9 (0‐36)	
Lymph node ratio (No. positive/No. resected)		
Median (Range)	0.08 (0–1)	
Unknown (no LN resected)	5	3.0
Tumor size (largest diameter) (cm)		
Median (Range)	3.0 (0.8‐18.0)	
Not Reported	18	10.9
Posterior margins involved		
No	102	61.8
Yes	36	21.8
Unknown	27	16.4
Type of adjuvant treatment		
No adjuvant treatment	76	46.1
Radiotherapy only	1	0.6
Chemotherapy only	33	20.0
Chemoradiotherapy	55	33.3
Pre‐op CEA (ng/mL)^a^		
Median (range)	3.3 (0.5‐61.8)	
Unknown	87	52.7
Post‐op CEA (ng/mL)^a^		
Median (range)	2.2 (0.7‐14.9)	
Unknown	124	75.2
Pre‐op CA19‐9 (U/mL)^a^ ^,^ ^b^		
Median (range)	187.0 (<0.6‐>10 000)	
Unknown	73	44.2
Post‐op CA19‐9 (U/mL)^a^ ^,^ ^b^		
Median (range)	25.2 (<0.6‐6825)	
Unknown	66	40.0
Pre‐op albumin (g/L)^a^		
Median (range)	34 (16‐48)	
Unknown	34	20.6
Post‐op albumin (g/L)^a^		
Median (range)	24 (14‐47)	
Unknown	26	15.8
Pre‐op neutrophil/lymphocyte ratio^a^		
Median (range)	2.9 (0.6‐36.5)	
Unknown	29	17.6
Post‐op neutrophil/lymphocyte ratio^a^		
Median (range)	12.3 (0.8‐49.6)	
Unknown	23	13.9

Abbreviation: NOS, Not otherwise specified.

^a^ Taken within 90 days before or after surgery.

^b^ Values of <0.6, < 2.0, > 5000, and >10 000 were taken as 0.6, 2.0, 5000, and 10 000, respectively, for the calculation of median.

### 3.2 Recurrence pattern

After median follow‐up of 15.5 months, 112 patients (67.9%) developed recurrence. The median time to recurrence was 8.87 months. 66.1% and 81.2% of patients developed recurrence at 12 and 24 months, respectively. (Figure S1).

Majority of patients developed distant recurrence as the first site of relapse. Seventy‐three (44.2%) had recurrence in a distant site, 20 (12.1%) had both local (defined as resection bed) and distant recurrences and 19 (11.5%) had solely local recurrence.

The most common site of recurrence was the liver (n = 58; 35.2%), followed by local recurrence (n = 39; 23.6%), distant lymph nodes (n = 31, 18.8%), peritoneum (n = 22, 13.3%), and lungs (n = 19; 11.5%).

### 3.3 Univariable analysis of OS

The median OS of the entire patient cohort was 19.7 months (95%CI: 16.9‐23.7). Median OS of patients who did not receive adjuvant therapy after curative resection was 15.7 months (95%CI: 11.7‐26.9). Median OS of patients who received adjuvant chemoradiotherapy or chemotherapy were 20.1 months (95%CI: 15.7‐28.2) and 23.8 months (95%CI: 19.1‐31.5) respectively. 1‐, 3‐, and 5‐year OS rates were 73.1% (95%CI: 65.1‐79.5), 28.0% (95%CI: 20.3‐36.1), and 14.8% (95%CI 7.6‐22.0), respectively.

Factors which conferred a poorer prognosis on OS by univariable analysis were: poorly differentiated/undifferentiated tumor (HR 2.15, 95% CI: 1.24‐3.74, *P* = .013), non‐pancreatic head tumors (HR 1.54, 95% CI: 1.04‐2.29, *P* = .037), N1 nodal status (HR 1.84, 95% CI: 1.24‐2.72, *P* = .002), lymph node ratio (LNR) of >0‐0.3 (HR 1.68, 95% CI: 1.09‐2.58, *P* = .001), LNR > 0.3 (HR 3.06, 95% CI: 1.75‐5.37, *P* = .001), presence of PNI (HR 2.62, 95% CI: 1.20‐5.73, *P* = .006), LVI (HR 1.52, 95% CI: 1.01‐2.29, *P* = .045), pre‐op CA 19‐9 (>75 U/mL) (HR 2.39, 95% CI 1.23‐4.63, *P* = .005), post‐op CA 19‐9 (>75 U/mL) (HR 2.61, 95% CI: 1.56‐4.38, *P* = .001). (Table 2).

**TABLE 2:** Univariable and multivariable analysis of overall survival

	No. of events/patients	Median OS, months (95% CI)	Log‐rank *P*‐value	Univariable		Multivariable	
				Hazard ratio (95% CI)	Cox model *P*‐value	Hazard ratio (95% CI)	Cox model *P*‐value
All patients	111/165	19.7 (16.9, 23.7)				98/146	
Age at surgery (years)							
<65	66/97	19.7 (16.9, 24.4)		1			
≥65	45/68	20.1 (14.1, 24.9)	.389	1.18 (0.81, 1.74)	.392		
Gender							
Male	59/91	19.7 (15.5, 24.4)		1			
Female	52/74	20.0 (16.8, 31.0)	.463	0.87 (0.60, 1.26)	.463		
Race							
Chinese	93/128	20.1 (17.4, 24.1)		1			
Non‐Chinese	18/36	13.5 (10.6, 31.5)	.198	1.40 (0.84, 2.33)	.216		
Smoking status							
Never	58/86	21.4 (17.9, 31.0)		1			
Former	21/30	15.7 (12.8, 19.6)		1.73 (1.04, 2.88)			
Current	6/10	31.6 (24.9, UD)	**.027**	0.58 (0.25, 1.36)	.032		
Alcohol consumption							
Never	64/96	20.0 (17.2, 28.2)		1			
Former	7/9	12.3 (6.9, 28.8)		1.70 (0.77, 3.73)			
Current	14/19	26.4 (16.9, 36.1)	.406	1.02 (0.57, 1.84)	.465		
Charlson comorbidities index							
1–2	46/63	19.7 (15.4, 28.8)		1			
>2	65/102	19.7 (15.7, 23.7)	.463	1.16 (0.78, 1.71)	.462		
Tumor site							
Head involved	74/121	21.1 (17.9, 24.9)		1		1	
Head not involved	37/44	15.4 (11.4, 24.4)	**.031**	1.54 (1.04, 2.29)	.037	1.59 (1.02, 2.48)	.046
AJCC TNM stage							
I	15/21	23.7 (11.4, 50.2)		1			
II	90/135	19.6 (15.7, 23.7)		1.16 (0.67, 2.02)			
III	6/9	26.9 (8.9, UD)	.730	0.89 (0.34, 2.31)	.719		
T stage							
T1/T2	26/34	19.7 (11.4, 30.2)		1			
T3/T4	85/131	19.7 (16.6, 24.4)	.505	0.86 (0.55, 1.34)	.511		
N stage							
N0	42/69	28.8 (20.4, 45.4)		1			
N1	68/95	15.5 (13.2, 19.7)	**.002**	1.84 (1.24, 2.72)	.002		
Histological grade							
Well/moderately differentiated	95/142	21.1 (17.4, 24.9)		1		1	
Poorly differentiated/Undifferentiated	15/21	11.2 (7.6, 20.0)	**.005**	2.15 (1.24, 3.74)	.013	1.86 (1.02, 3.38)	.058
Type of surgery							
Whipples operation or PPPD	77/121	20.1 (17.4, 24.1)		1			
Pancreatectomy, distal or subtotal	29/37	17.6 (11.4, 31.6)		1.29 (0.84, 1.98)			
Pancreatectomy, total	3/4	4.3 (3.1, UD)		7.24 (2.22, 23.60)			
Pancreatectomy, NOS	2/3	14.2 (14.2, UD)	**.002**	1.31 (0.32, 5.37)	.057		
Resection margins							
R0	52/80	19.7 (16.9, 26.9)		1			
R1	59/85	19.7 (14.2, 24.1)	.612	1.10 (0.76, 1.60)	.611		
Perineural invasion							
No	7/14	50.2 (17.2, UD)		1		1	
Yes	94/135	19.1 (15.5, 22.6)	**.013**	2.62 (1.20, 5.73)	.006	2.36 (1.07, 5.23)	.018
Lymphovascular invasion							
No	54/80	23.7 (17.7, 35.4)		1			
Yes	42/62	16.6 (11.7, 20.1)	**.042**	1.52 (1.01, 2.29)	**.045**		
Lymph node ratio							
0	38/64	31.0 (20.1, 45.4)		1		1	
>0–0.3	49/70	17.9 (14.1, 22.0)		1.68 (1.09, 2.58)		1.58 (1.00, 2.49)	
>0.3	20/26	12.3 (7.5, 19.6)	<.001	3.06 (1.75, 5.37)	.001	3.36 (1.83, 6.16)	.001
Tumor size (largest diameter) (cm)							
≤3	50/78	23.7 (17.9, 28.8)		1			
>3	51/69	14.1 (11.5, 21.1)	.017	1.61 (1.09, 2.38)	**.018**		
Posterior margins involved							
No	68/102	19.7 (15.7, 26.4)		1			
Yes	24/36	18.5 (10.8, 31.6)	.797	1.06 (0.67, 1.70)	.798		
Adjuvant treatment							
None	48/76	15.7 (11.7, 26.9)		1			
Chemotherapy only	19/33	23.8 (19.1, 31.5)		0.74 (0.43, 1.26)			
Chemoradiotherapy	43/55	20.1 (15.7, 28.2)	.528	0.89 (0.59, 1.35)	.520		
Pre‐op CEA (ng/ml)							
≤5	27/44	22.0 (17.6, 44.6)		1			
>5	28/34	14.1 (10.0, 24.4)	.110	1.54 (0.90, 2.61)	.114		
Post‐op CEA (ng/ml)							
≤5	27/34	21.8 (14.7, 30.2)		1			
>5	6/7	21.4 (3.1, UD)	.330	1.55 (0.64, 3.80)	.356		
Pre‐op CA19‐9 (U/ml)							
≤75	13/28	55.5 (14.0, 74.4)		1			
>75	51/64	19.1 (15.3, 22.0)	.008	2.39 (1.23, 4.63)	.005		
Post‐op CA19‐9 (U/ml)							
≤75	48/72	22.6 (18.5, 30.2)		1			
>75	22/27	13.2 (8.4, 19.4)	<.001	2.61 (1.56, 4.38)	**.001**		
Pre‐op albumin (g/L)							
>35	38/54	22.0 (14.2, 31.6)		1			
≤35	50/77	17.9 (14.1, 23.7)	.870	1.04 (0.68, 1.58)	.869		
Post‐op albumin (g/L)							
>35	11/17	24.4 (17.6, 36.0)		1			
≤35	83/122	18.5 (14.7, 22.6)	.300	1.39 (0.74, 2.62)	.283		
Pre‐op NLR							
≤5	77/109	19.1 (15.4, 26.4)		1			
>5	16/27	19.4 (12.8, 24.1)	.363	1.29 (0.74, 2.24)	.377		
Post‐op NLR							
≤5	14/18	22.6 (13.2, 50.0)		1			
>5	83/124	19.4 (15.4, 24.1)	.861	1.05 (0.60, 1.86)	.861		

Abbreviations: NLR, neutrophil‐lymphocyte ratio; PPPD, pylori preserving pancreaticoduodenectomy; UD, undefined.

*Note*: For the multivariable analysis, only variables with less than 10% missing data were considered in the forward selection procedure. The criterion for variable addition was *P* < .10.

### 3.4 Multivariable analysis of OS

**FIGURE 1:**
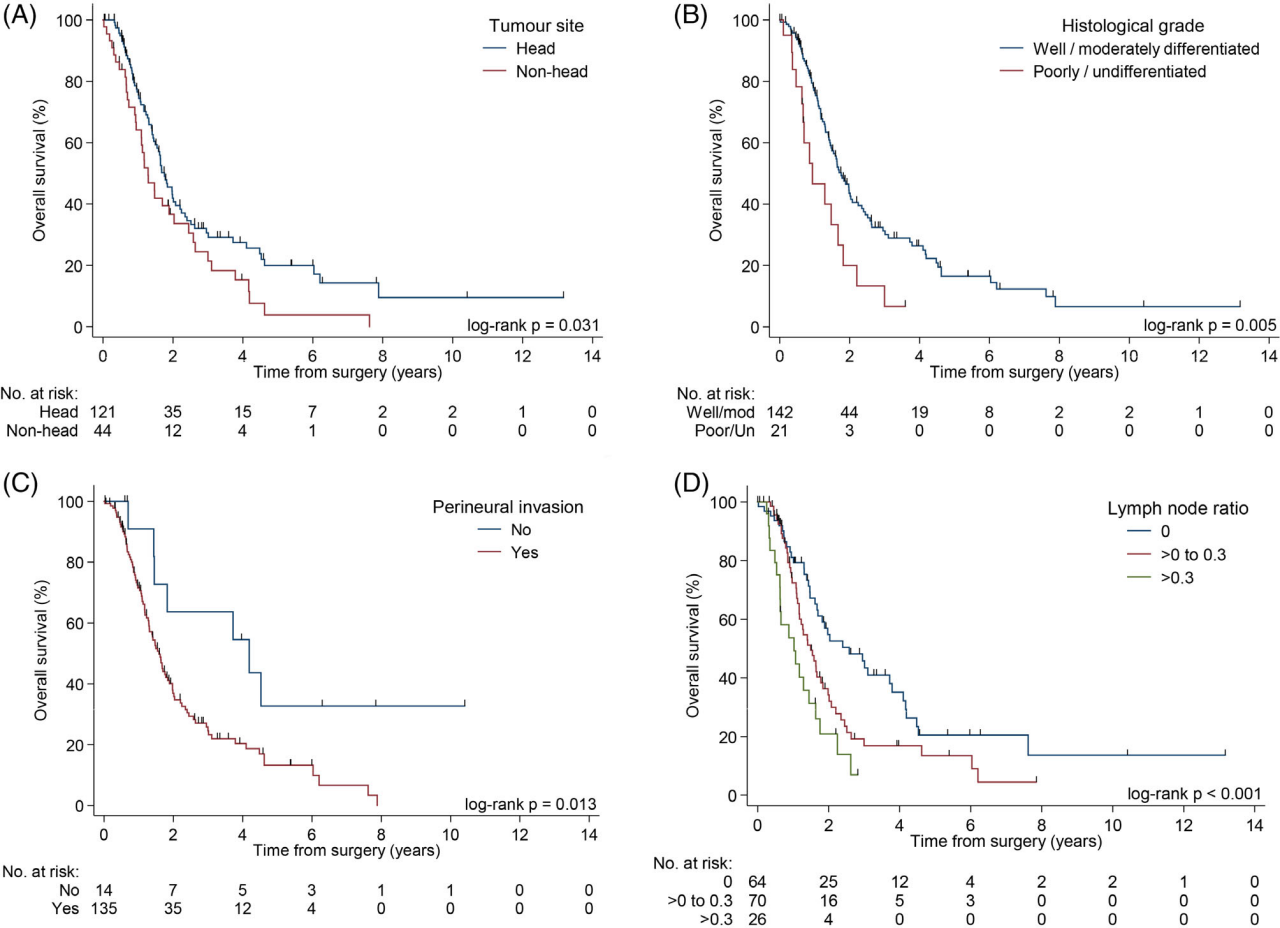
Kaplan‐Meier curves of OS by tumor site, A, tumor grade, B, PNI, C, LNR, D

The final multivariable model for OS revealed that LNR > 0‐0.3 (HR  1.58, 95%CI:  1.00‐2.49, *P* < .001), lymph node ratio > 0.3‐1 (HR  3.36, 95%CI:  1.83‐6.16, *P* = .001), non‐pancreatic head tumors (HR  1.59, 95%CI:  1.02‐3.38, *P* = .046), presence of PNI (HR  2.36 95%CI:  1.07‐5.23, *P* = .018), and poorly differentiated or undifferentiated tumor grade (HR  1.86, 95%CI:  1.02‐3.38, *P* = .058) were negative predictors of survival. (Table 2) The Kaplan‐Meier plot of the OS for the above‐mentioned prognostic factors can be found in Figure 1.

## 4 DISCUSSION

The median survival of patients in this study was 19.7 months (95%CI: 16.9‐23.7) with a 5‐year OS of 14.8% (95%CI: 7.6‐22). This is comparable to the experience of major centers in both Western and Asian series with a median survival ranging from 13 to 24 months and a 5‐year OS ranging from 4% to 27%. The patient characteristics and prognostics factors described in both Western and Asian series are also similar (Table 3).

**TABLE 3:** Series of resected pancreatic adenocarcinoma in Asian and Western Centers

Series (Author, country)	Year	Number of Patients (N)	Median age	Gender (%)	Tumour site (%)	Stage (%)	Tumour	Lymph node (%)	Differentiation (%)	Adjuvant tx (%)	Median OS (mth)	1‐year OS (%)	3‐year OS (%)	5‐year OS (%)	Prognostic Factors (multivariable analysis)
Asian															
Liu et al, China^22^	2007‐2015	1223	62	M: 57	Head/Body: 56.9 Tail: 43.1	IA: 9.4 IB: 30.9 IIA: 15.8 IIB: 33.7 III: 10.2	Mean: 3.77 cm	N1: 43.9	WD: 65.0 MD/PD: 35.0	82.5	18.7	64.0	28.7	NR	Tumor grade Post‐operative (post‐op) CA 19–9
Yamamoto et al, Japan^23^	2001‐2015	100	70	M: 58	NR	IA: 5 IB: 3 IIA: 38 IIB 52 III: 1 IV: 1	Median: 2.6 cm	N1: 53	NR	64.0	NR	79.7	40.9	22.6	NR
You et al, Korea^5^	2005‐2017	351	63.3	M: 57.8	Head: 64.1 Non‐head: 32.5 Overlapping: 3.4	I: 19.1 II: 67.0 III: 12.8 IV: 1.1	NR	N1: 57.3	WD: 8.0 MD: 82.3 PD: 8.8 UD: 0.9	100.0^a^	31.7	NR	NR	NR	LNR Tumor grade Lymphovascular invasion (LVI) Perineural invasion (PNI) Tumor stage (T‐stage) Pre‐operative (pre‐op) CA 19‐9
Xu et al, China^24^	2010‐2014	353	61	M: 56.9	Head/body: 57.5 Tail: 42.5	I: 24.4 IIA: 29.7 IIB: 45.9	Mean: 4.13 cm	N1: 45.9	WD/MD: 63.7 PD: 36.3	Chemo (82.4) ChemoRT (31.7)	18.1	62.2	27.1	‐	Those with elevated post‐op serum CA19‐9: Tumour size, no adjuvant chemoradiotherapy (chemoRT), post‐op CA125, no decrease in CA19‐9 from pre‐op Those with normal post‐op serum CA19‐9: No adjuvant chemoradiotherapy, post‐op CA125, post‐op CEA
Haruki et al, Japan^25^	2001‐2011	113	66.8 (mean)	M: 61.9	NR	0:6.2 I: 2.7 II: 16.8 III: 48.7 IV: 25.7	NR	NR	NR	NR	NR	NR	NR	24.1	CRP/albumin ratio Higher TNM stage
Shin et al, Korea^26^	2000‐2007	528	61	M: 60.2	Head: 74.4 Non‐head: 25.6		≤3 cm: 51.3 >3 cm: 48.7	N1: 42.0	WD/MD: 78.0 PD: 15.2 Missing 6.8	NR	NR	NR	NR	15.5	Tumor size Tumor grade Node‐stage (N‐stage) PNI LVI Portal/mesenteric vein invasion
Western															
Sohn et al, USA^27^	1984‐1999	616	64.3 (mean)	M: 54	NR	NR	Mean: 3.2 cm	N1: 72.0	WD/MD: 64.0 PD: 36.0	74.0	17.0	63.0	25.0	17.0	Resection Margin Tumor size Intra‐op blood loss Tumor grade Post‐op chemoRT
Katz et al, USA^10^	1990‐2002	329	64	M: 58	Head: 92 Non‐head: 8	NR	Mean: 3.0 cm	N1: 52.0	NR	91.0	24.0	NR	NR	27.0	N‐stage Prior attempts at resection
Schnelldorfer et al, USA^28^	1981‐2001	357	65 (mean)	M: 54	Head: 100	IA: 7.6 IB: 14.9 IIA: 27.4 IIB: 48.5 III: 0.8 IV: 0.8	Mean: 3.2 cm	N1: 49.4	WD: 0 MD: 21.9 PD: 55.8 UD: 22.3	77.0	17.0	NR	NR	18.0	Tumour size N‐stage
Winter et al, USA^29^	1990‐1999	399	NR	NR	NR	NR	NR	NR	NR	NR	25.6	68.0	NR	20.0	‐
Winter et al, USA^29^	2000‐2009	625	NR	NR	NR	NR	NR	NR	NR	NR	24.5	68.0	NR	8.0	‐
Lewis et al, USA^6^	2001–2011	424	67	M: 50.5	NR	IA: 3.8 IB: 6.8 IIA: 19.3 IIB 64.9 III: 2.4 IV: 2.6	NR	N1: 68.4	WD: 10.8 MD: 49.5 PD 39.7	76.4	21.3	76.0	34.0	23.0	T‐stage N‐stage LNR Tumor size Tumor grade LVI PNI Resection margin Adjuvant treatment Pre‐op physiology
Konstantinidis et al, USA^30^	1993‐2008	517	67	M: 47.2	NR	NR	Median: 3.0 cm	N1: 31.5	WD: 3.5 MD: 54.5 PD: 39.7 UD: 2.3	NR	19.7	NR	NR	17	Size of tumour Tumor grade LVI PNI Resection margin LNR

Abbreviation: CRP, C‐reactive protein; LNR, Lymph Node Ratio; LVI, Lymphovascular invasion; M, Male; MD, Moderately differentiated; NR, Not reported; PD, Poorly differentiated; PNI, Perineural invasion; UD, Undifferentiated; WD, Well differentiated

a Only patients who received adjuvant chemotherapy are included in this study.

Despite the benefits of adjuvant chemotherapy, only 50.9% of our patients received adjuvant treatment, which was comparable with other institutions and large series reporting rates of approximately 35% to 60%.^31^, ^32^, ^33^, ^34^ There are numerous reasons why patients do not receive adjuvant chemotherapy. These include post‐operative complications leading to poor performance status post‐surgery, tumor recurrence or metastases detected prior to initiation of adjuvant chemotherapy, and patient's preferences.^35^, ^36^, ^37^ Patients who received adjuvant chemotherapy in our series had an OS of 23.8 months as compared to 15.7 months for those who did not receive adjuvant chemotherapy. This is comparable to that of the Phase 3 trials evaluating the efficacy of these regimes,^11^, ^12^, ^14^, ^38^ and consistent with real‐world data described by other authors.^34^, ^37^ Given the low rates of receipt of adjuvant chemotherapy and early dissemination of disease in PDAC, a neoadjuvant approach may be advantageous.^39^, ^40^ Studies exploring this approach have conflicting results. The Phase 3 PREOPANC‐1 trial randomized patients to preoperative chemoradiotherapy followed by surgery and four courses of adjuvant gemcitabine or to immediate surgery and six courses of adjuvant gemcitabine. There was no difference in the OS by intention to treat in both groups.^63^ The Prep‐02/JSAP‐05 randomized Phase 2/3 trial randomized 362 patients with resectable PDAC to neoadjuvant gemcitabine and S‐1 followed by surgery and adjuvant S‐1 or initial surgery and adjuvant S‐1. There was a significant benefit of neoadjuvant gemcitabine and S‐1 followed by surgery and adjuvant S‐1 compared with initial surgery and adjuvant S‐1 therapy (median OS: 36.7 vs 26.6 months, HR 0.72 (95%CI: 0.55‐0.94), *P* = .015).^64^ However, this was done exclusively in Japanese patients and the generalizability of these data is debatable. The SWOG S1505 Phase 2 randomized trial randomized patients with resectable PDAC to perioperative FOLFIRINOX or perioperative gemcitabine and nab‐paclitaxel. The primary outcome was 2‐year OS. Each arm was compared against the historical threshold of 40%. The 2‐year OS was 41.6% with mFOLFIRINOX (*P* = .42) and 48.8% with gemcitabine/nab‐paclitaxel (*P* = .12).^65^ There are multiple other trials examining this question including the randomized Phase 2/3 NEPAFOX trial (ClinicalTrials.gov identifier: NCT02172976) which is evaluating neoadjuvant FOLFIRINOX, surgery, and adjuvant FOLFIRINOX compared with surgery and adjuvant gemcitabine in patients with resectable and borderline resectable pancreatic cancer. There is also the randomized Phase 2 NEONAX trial (ClinicalTrials.gov identifier: NCT02047513) which compares neoadjuvant gemcitabine and nab‐paclitaxel followed by surgery and adjuvant gemcitabine and nab‐paclitaxel compared with initial surgery and adjuvant gemcitabine and nab‐paclitaxel. While no patients in our series received neoadjuvant treatment, it is a promising approach worth considering and we await the results of ongoing trials.

The pattern of recurrence in our series of patients is similar to that reported in the literature.^10^ Most of the recurrences occurred within the first year after surgery as demonstrated in Figure S1. The most common sites of recurrence are the liver, local recurrence, distant lymph nodes, lungs, and peritoneum. 61.3% of patients in our study developed recurrence within 1 year after curative resection; this is reflective of the aggressive disease biology and presence of micrometastases at diagnosis.

In this study consisting of Asian patients, we identified four prognostic factors associated with poor prognosis: LNR > 0.3, poorly differentiated/undifferentiated tumor grade, location of tumor at the body or tail and the presence of PNI.

LNR has been found to be an independent prognostic factor in various studies.^5^, ^41^, ^42^ Different groups have used different cutoffs for the LNR. Valsangkar et al demonstrated that increasing values of LNR of 0.2, 0.20 to 0.30 and ≥0.30 were associated with poor prognosis,^41^ Huebner et al showed that a LNR of ≥0.17 had poorer prognosis.^42^ We found that a LNR ≥0.30 was associated with a poorer prognosis. Patients with LNR of 0, >0 to 0.3 and > 0.3 had median OS of 31.0, 17.9, and 12.3 months, respectively. Total number of lymph nodes examined (TLN) may be of prognostic significance, especially in patients with pN0 disease. Slidell et al found that patients with pN0 disease could be further stratified based on the number of lymph nodes evaluated, with those with 11 or less LN examined having a poorer prognosis.^43^ Another study showed that those with <12 TLN had a poorer prognosis, but this did not reach statistical significance.^44^ In our study, however, we did not find that the TLN was a prognostic factor in patients with pN0 disease or in our entire cohort. While nodal status is incorporated as a stratification in a large proportion of randomized adjuvant trials in pancreatic cancer,^12^, ^13^, ^15^, ^18^ LNR could be a better stratification factor. LNR did not feature as a stratification factor in any of the randomized trials (Table 4). The only randomized trial, which included LNR in its patients' clinic‐pathological characteristics, was JASPAC‐01 trial.^15^ Tumor grade is a known prognostic factor found in many studies, including various RCTs.^5^, ^6^, ^11^, ^14^, ^18^, ^22^, ^26^, ^27^, ^30^, ^48^, ^49^ (Tables 3 and 4) Our study confirmed this finding. While Brennan et al found that tumors located at the head are associated with a worse prognosis, our results are contrary to this.^50^ We found that patients with tumors at the body or tail had poorer prognosis. Multiple studies have suggested that the anatomical site is a prognostic factor; however, studies have been conflicting regarding which site is associated with a better prognosis.^51^, ^52^, ^53^, ^54^ Artinyan et al and Watanabe et al reported that patients with body/tail PDAC are more likely to be have unresectable or metastatic disease at presentation and consequently have poorer OS. This is attributed to the earlier onset of symptoms (eg, jaundice) in patients with head lesions.^52^, ^53^ Body/tail lesions were found to be a poorer prognostic factor compared with head lesions even in patients who had undergone surgical resection.^53^ This may potentially be due to more aggressive tumor biology for lesions arising from the body/tail.^55^ However, Lau et al, which utilized the Surveillance, Epidemiology, and End Results (SEER) registry, found that patients with local‐stage pancreatic body/tail cancer had higher OS compared with local‐stage pancreatic head cancer.^51^

**TABLE 4:** Phase 3 randomized clinical trials evaluating efficacy of adjuvant treatment in resected pancreatic adenocarcinoma

Randomized controlled trials	Arms	N	Stratifications	Clinico‐pathological features described in patient characteristics Y: Yes, N: No												Median OS (mths)	5‐year‐OS (%)
				Age	Gender	T‐status	Nodal status	LNR	Resection status	Grade	Baseline CA19‐9	Post‐operative CA19‐9	Site of primary	PNI	LVI		
ESPAC 1^11^	Observation (Obs) 5‐FU ChemoRT ChemoRT followed by 5‐FU	289	Country Resection margin	Y	Y	Y	Y	N	Y	Y	N	N	N	N	N	Obs: 16.9 5‐FU: 20.1 ChemoRT: 15.9 ChemoRT followed by 5‐FU: 19.9	Obs: 11 5‐FU: 29 ChemoRT: 7 ChemoRT followed by 5‐FU: 13
CONKO‐001^12^	Gemcitabine (Gem) Observation	368	Tumour stage: T1‐2 vs T3‐4 Nodal status: N0 vs N1 Resection margin: R0 vs R1	Y	Y	Y	Y	N	Y	Y	N	N	N	N	N	Gem: 22.8 Obs: 20.2	Gem: 20.7 Obs: 10.4
RTOG 9704^13^, ^45^	5‐FU‐RT Gem‐RT	451	Tumor diameter: <3 cm vs ≥3 cm Nodal status: N0 vs N1 Surgical margins: R0 vs R1 vs unknown	Y	Y	Y	Y	N	Y	Y	Y	N	Y	N	N	5‐FU‐RT: 16.9 Gem‐RT: 20.6	5FU: 18 Gem: 22
ESPAC 3^14^	Gem 5‐FU	1008	Country Surgical margins: R0 vs R1	Y	Y	Y	Y	N	Y	Y	N	Y	N	N	N	Gem:23.6 5‐FU:23.0	‐
JASPAC‐01^15^	Gem TS‐one	377	Study site Surgical margin: R0 vs R1 Nodal status: N0 vs N1	Y	Y	Y	Y	Y	Y	N	Y	N	N	N	N	Gem:25.5 TS‐one: 46.5	Gem: 24.4 TS‐one: 44.1
ESPAC‐4^38^, ^46^N	Gem Gem/Cape	730	Country R0 vs R1	Y	Y	Y	Y	N	Y	Y	Y	Y	N	N	N	Gem: 25.5 Gem/Cape: 28.0	Gem: 20.0 Gem/Cape: 28.0
PRODIGE‐24^18^	Gem mFFX	493	Study site Surgical margin: R0 vs R1 Nodal status: N0 vs N1 Post‐op CA19‐9 (≤90 U/mL vs 91‐180 U/mL)	Y	Y	Y	N	N	Y	Y	N	Y	Y	Y	Y	Gem: 35.0 mFFX: 54.4	‐
APACT^17^, ^47^	Gem Gem/nab‐paclitaxel	866	Country Surgical margin: R0 vs R1 Nodal status: N0 vs N1	Y	Y	Y	Y	N	Y	Y	Y	N	N	N	N	Gem: 37.7 Gem/nab‐paclitaxel: 41.8	‐

Abbreviations: 5‐FU, 5‐Flurouracil; Cape, Capecitabine; Gem, Gemcitabine; mFOLFIRINOX, modified 5‐FU, leucovorin, oxaliplatin, irinotecan; nab‐Paclitaxel, nanoparticle albumin‐bound paclitaxel; Obs, Observation; TS‐one, tegafur, gimeracil, oteracil.

Chatterjee et al found that the presence of PNI and LVI correlated with poorer outcomes. We found that the presence of PNI but not LVI was associated with poor prognosis. PNI is the presence of cancer cells along nerves and/or within the epineurial, perineurial, and endoneurial spaces of the neuronal sheath and is commonly found in PDAC.^56^ The presence of PNI has been demonstrated as a negative prognostic factor in multiple studies.^5^, ^6^, ^26^, ^30^ (Table 3).

While the previously described factors are well described in the literature to be prognostic, the prognostic value of the resection margin remains controversial.^57^

Margin status has been identified as prognostic factor in multiple studies.^58^, ^59^ However, other studies have demonstrated no relationship between the resection margin and OS.^60^, ^61^ Conflicting results have also been found for the posterior resection margin.^58^, ^62^ Our study found that resection margin status (R0 vs R1) and the posterior resection margin status (R0 vs R1) were not independently associated with OS in the multivariable analysis. There are numerous postulations for the conflicting results. First, the definition of microscopic margin positivity differs from study to study.^19^, ^60^ Second, there are wide variability in the way different centers handle and sample the resection tissue.^57^ Third, the definition of the posterior margin is also not standardized in multiple studies.^57^

Taking the above together, our study showed that our cohort had similar prognostic factors, recurrence patterns, and survival as other Western and Asian institutions.^5^, ^6^, ^10^, ^22^, ^23^, ^24^, ^25^, ^26^, ^27^, ^28^, ^29^, ^30^ (Table 3) In the APACT trial which recruits both Western and Asian patients, country was used as a stratification factor.^17^ Given the similarity in clinical characteristics in Western and Asian patients with PDAC, using country as a stratification factor may not be necessary. On the other hand, LNR and presence of PNI have consistently been found to be a significant prognostic factor in RCTs or large series from high‐volume centres^5^, ^6^, ^11^, ^14^, ^16^, ^18^, ^22^, ^26^, ^27^, ^30^, ^48^, ^49^ (Tables 3 and 4) and should perhaps be used as a stratification factor instead.

Our study has several limitations. While we managed to demonstrate applicability of adjuvant therapy in a general Asian population consistent with what has been reported in RCT, all the patients in this cohort received single agent systemic therapy (gemcitabine or 5FU). A number of RCT has since been reported providing evidence for doublet and triplet combination therapies.^17^, ^18^ Future population‐based studies are needed to clarify its applicability to a general population. As this study is retrospective in nature, there may be recall bias. Furthermore, the study sample size is modest, perhaps explaining for lack of statistical significance in previously reported prognostic factors (eg, resection margins and presence of LVI). Finally, incomplete capture of variables may introduce bias in survival analysis.

In conclusion, the survival of Asian patients with resected PDAC who received adjuvant chemotherapy is comparable to reported randomized trials. Clinical characteristics of Asian patients with resected PDAC are similar to datasets described among patients from the West. Hence, geographical locations/country of origin may not be a necessary stratification factor in RCTs. Conversely, LNR and status of PNI ought to be incorporated.

## CONFLICT OF INTEREST

Su Pin Choo has received research funding and speaking fees from Bristol‐Myers Squibb (BMS) speaking fees from Lilly, research funding from Sirtex, and has participated on advisory boards for BMS, Sirtex, Lilly, Norvatis, Eisai, Bayer, Celgene. David Tai has received research funding for BMS and Sirtex, honorarium from Bayer and has participated on advisory boards for Eisai, Bayer, and Ipsen. Joycelyn Jie Xin Lee has received research funding from Bayer, honorarium from BMS and Ipsen, and has participated on advisory boards for Bayer and Ipsen.

## AUTHOR CONTRIBUTIONS

All authors had full access to the data in the study and take responsibility for the integrity of the data and the accuracy of the data analysis. Conceptualization, K.Y.Y.N., E.W.X.C., D.T.; Methodology, K.Y.Y.N., E.W.X.C., D.T.; Investigation, K.Y.Y.N., E.W.X.C., B.J.; Formal Analysis, K.Y.Y.N., E.W.X.C., C.L.; Resources, D.T.; Writing ‐ Original Draft, K.Y.Y.N., E.W.X.C., D.T.; Writing ‐ Review & Editing, All authors: Visualization, K.Y.Y.N., E.W.X.C., D.T.

## ETHICAL STATEMENT

Our study was approved by the Centralized Institutional Review Board of our institution.

## Supporting information


**Figure S1.** Recurrence pattern.

## Data Availability

The unidentified dataset is available upon reasonable requests made to the corresponding author.

## References

[1] Jemal A , Bray F , Center MM , Ferlay J , Ward E , Forman D . Global cancer statistics. CA Cancer J Clin. 2011;61(2):69‐90. 21296855 10.3322/caac.20107

[2] Allen PJ , Kuk D , Castillo CF , et al. Multi‐institutional validation study of the American joint commission on cancer (8th edition) changes for T and N staging in patients with pancreatic adenocarcinoma. Ann Surg. 2017;265(1):185‐191. 27163957 10.1097/SLA.0000000000001763 PMC5611666

[3] Chun YS , Pawlik TM , Vauthey JN . 8th edition of the AJCC cancer staging manual: pancreas and hepatobiliary cancers. Ann Surg Oncol. 2018;25(4):845‐847. 28752469 10.1245/s10434-017-6025-x

[4] Stotz M , Gerger A , Eisner F , et al. Increased neutrophil‐lymphocyte ratio is a poor prognostic factor in patients with primary operable and inoperable pancreatic cancer. Br J Cancer. 2013;109(2):416‐421. 23799847 10.1038/bjc.2013.332 PMC3721392

[5] You MS , Lee SH , Choi YH , et al. Lymph node ratio as valuable predictor in pancreatic cancer treated with R0 resection and adjuvant treatment. BMC Cancer. 2019;19(1):952. 31615457 10.1186/s12885-019-6193-0 PMC6794802

[6] Lewis R , Drebin JA , Callery MP , et al. A contemporary analysis of survival for resected pancreatic ductal adenocarcinoma. HPB. 2013;15(1):49‐60. 23216779 10.1111/j.1477-2574.2012.00571.x PMC3533712

[7] Butturini G , Stocken DD , Wente MN , et al. Influence of resection margins and treatment on survival in patients with pancreatic cancer: meta‐analysis of randomized controlled trials. Arch Surg. 2008;143(1):75‐83.discussion 83. 18209156 10.1001/archsurg.2007.17

[8] Chang DK , Johns AL , Merrett ND , et al. Margin clearance and outcome in resected pancreatic cancer. J Clin Oncol. 2009;27(17):2855‐2862. 19398572 10.1200/JCO.2008.20.5104

[9] McGuigan A , Kelly P , Turkington RC , Jones C , Coleman HG , McCain RS . Pancreatic cancer: a review of clinical diagnosis, epidemiology, treatment and outcomes. World J Gastroenterol. 2018;24(43):4846‐4861. 30487695 10.3748/wjg.v24.i43.4846 PMC6250924

[10] Katz MHG , Wang H , Fleming JB , et al. Long‐term survival after multidisciplinary management of resected pancreatic adenocarcinoma. Ann Surg Oncol. 2009;16(4):836‐847. 19194760 10.1245/s10434-008-0295-2 PMC3066077

[11] Neoptolemos JP , Stocken DD , Friess H , et al. A randomized trial of chemoradiotherapy and chemotherapy after resection of pancreatic cancer. N Engl J Med. 2004;350(12):1200‐1210. 15028824 10.1056/NEJMoa032295

[12] Oettle H , Neuhaus P , Hochhaus A , et al. Adjuvant chemotherapy with gemcitabine and long‐term outcomes among patients with resected pancreatic cancer: the CONKO‐001 randomized trial. JAMA. 2013;310(14):1473‐1481. 24104372 10.1001/jama.2013.279201

[13] Regine WF , Winter KA , Abrams R , et al. Fluorouracil‐based chemoradiation with either gemcitabine or fluorouracil chemotherapy after resection of pancreatic adenocarcinoma: 5‐year analysis of the U.S. intergroup/RTOG 9704 phase III trial. Ann Surg Oncol. 2011;18(5):1319‐1326. 21499862 10.1245/s10434-011-1630-6 PMC3548408

[14] Neoptolemos JP , Stocken DD , Bassi C , et al. Adjuvant chemotherapy with fluorouracil plus Folinic acid vs gemcitabine following pancreatic cancer resection: a randomized controlled trial. JAMA. 2010;304(10):1073‐1081. 20823433 10.1001/jama.2010.1275

[15] Uesaka K , Boku N , Fukutomi A , et al. Adjuvant chemotherapy of S‐1 versus gemcitabine for resected pancreatic cancer: a phase 3, open‐label, randomised, non‐inferiority trial (JASPAC 01). Lancet. 2016;388(10041):248‐257. 27265347 10.1016/S0140-6736(16)30583-9

[16] Neoptolemos JP , Palmer DH , Ghaneh P , et al. ESPAC‐4: a multicenter, international, open‐label randomized controlled phase III trial of adjuvant combination chemotherapy of gemcitabine (GEM) and capecitabine (CAP) versus monotherapy gemcitabine in patients with resected pancreatic ductal adenocarcinoma: five year follow‐up. J Clin Oncol. 2020;38(15_suppl):4516‐4516.

[17] Reni M , Riess H , O'Reilly EM , et al. Phase III APACT trial of adjuvant nab‐paclitaxel plus gemcitabine (nab‐P + gem) versus gemcitabine (gem) alone for patients with resected pancreatic cancer (PC): outcomes by geographic region. J Clin Oncol. 2020;38(15_suppl):4515‐4515.

[18] Conroy T , Hammel P , Hebbar M , et al. FOLFIRINOX or gemcitabine as adjuvant therapy for pancreatic cancer. N Engl J Med. 2018;379(25):2395‐2406. 30575490 10.1056/NEJMoa1809775

[19] Campbell F , Foulis AK , Verbeke CS . Dataset for the histopathological reporting of carcinomas of the pancreas, ampulla of Vater and common bile duct. 2 Carlton House Terrace, London, SW1Y 5AF: The Royal College of Pathologists; 2010. https://www.rcpath.org/uploads/assets/34910231‐c106‐4629‐a2de9e9ae6f87ac1/G091‐Dataset‐for‐histopathological‐reporting‐of‐carcinomas‐of‐the‐pancreas‐ampulla‐of‐Vater‐and‐common‐bile‐duct.pdf

[20] Goh BKP , Tan Y‐M , Chung Y‐FA , et al. Critical appraisal of 232 consecutive distal pancreatectomies with emphasis on risk factors, outcome, and Management of the Postoperative Pancreatic Fistula: a 21‐year experience at a single institution. Arch Surg. 2008;143(10):956‐965. 18936374 10.1001/archsurg.143.10.956

[21] Goh BKP , Tan YM , Cheow PC , et al. Outcome of distal pancreatectomy for pancreatic adenocarcinoma. Dig Surg. 2008;25(1):32‐38. 18292659 10.1159/000117821

[22] Liu L , Xu HX , He M , et al. A novel scoring system predicts postsurgical survival and adjuvant chemotherapeutic benefits in patients with pancreatic adenocarcinoma: implications for AJCC‐TNM staging. Surgery. 2018;163(6):1280‐1294. 29548773 10.1016/j.surg.2018.01.017

[23] Yamamoto T , Uchida Y , Terajima H . Clinical impact of margin status on survival and recurrence pattern after curative‐intent surgery for pancreatic cancer. Asian J Surg. 2019;42(1):93‐99. 29249392 10.1016/j.asjsur.2017.09.003

[24] Xu HX , Liu L , Xiang JF , et al. Postoperative serum CEA and CA125 levels are supplementary to perioperative CA19‐9 levels in predicting operative outcomes of pancreatic ductal adenocarcinoma. Surgery. 2017;161(2):373‐384. 27838102 10.1016/j.surg.2016.08.005

[25] Haruki K , Shiba H , Shirai Y , et al. The C‐reactive protein to albumin ratio predicts long‐term outcomes in patients with pancreatic cancer after pancreatic resection. World J Surg. 2016;40(9):2254‐2260. 26956901 10.1007/s00268-016-3491-4

[26] Shin SH , Kim SC , Hong SM , et al. Can statistically determined prognostic factors predict the long‐term survival of patients with pancreatic ductal adenocarcinoma following surgical resection?: clinicopathological analysis of 82 long‐term survivors. Pancreas. 2014;43(4):571‐577. 24681875 10.1097/MPA.0000000000000063

[27] Sohn TA , Yeo CJ , Lillemoe KD , et al. Resected adenocarcinoma of the pancreas ‐ 616 patients: results, outcome and prognostic indicators. Gastroenterology. 2000;118(4, Part 1):A1059. 10.1016/s1091-255x(00)80105-5 11307091

[28] Schnelldorfer T , Ware AL , Sarr MG , et al. Long‐term survival after pancreatoduodenectomy for pancreatic adenocarcinoma. Ann Sur. 2008;247(3):456‐462. 10.1097/sla.0b013e3181613142. 18376190

[29] Winter JM , Brennan MF , Tang LH , et al. Survival after resection of pancreatic adenocarcinoma: results from a single institution over three decades. Ann Surg Oncol. 2012;19(1):169‐175. 21761104 10.1245/s10434-011-1900-3

[30] Konstantinidis IT , Deshpande V , Zheng H , et al. Does the mechanism of lymph node invasion affect survival in patients with pancreatic ductal adenocarcinoma? J Gastrointest Surg. 2010;14(2):261‐267. 19937477 10.1007/s11605-009-1096-z PMC3135335

[31] Parmar AD , Vargas GM , Tamirisa NP , Sheffield KM , Riall TS . Trajectory of care and use of multimodality therapy in older patients with pancreatic adenocarcinoma. Surgery. 2014;156(2):280‐289. 24851723 10.1016/j.surg.2014.03.001 PMC4099282

[32] Hsu CC , Herman JM , Corsini MM , et al. Adjuvant chemoradiation for pancreatic adenocarcinoma: the Johns Hopkins Hospital‐Mayo Clinic collaborative study. Ann Surg Oncol. 2010;17(4):981‐990. 20087786 10.1245/s10434-009-0743-7 PMC2840672

[33] Kutlu OC , Vega EA , Salehi O , et al. Laparoscopic pancreatectomy for cancer in high volume centers is associated with an increased use and fewer delays of adjuvant chemotherapy. HPB. 2020.(23 .(4):625‐632. 32988752 10.1016/j.hpb.2020.09.003

[34] Altman AM , Wirth K , Marmor S , et al. Completion of adjuvant chemotherapy after upfront surgical resection for pancreatic cancer is uncommon yet associated with improved survival. Ann Surg Oncol. 2019;26(12):4108‐4116. 31313044 10.1245/s10434-019-07602-6 PMC11982014

[35] Merkow RP , Bilimoria KY , Tomlinson JS , et al. Postoperative complications reduce adjuvant chemotherapy use in resectable pancreatic cancer. Ann Surg. 2014;260(2):372‐377. 24374509 10.1097/SLA.0000000000000378

[36] Labori KJ , Katz MH , Tzeng CW , et al. Impact of early disease progression and surgical complications on adjuvant chemotherapy completion rates and survival in patients undergoing the surgery first approach for resectable pancreatic ductal adenocarcinoma ‐ a population‐based cohort study. Acta Oncol. 2016;55(3):265‐277. 26213211 10.3109/0284186X.2015.1068445

[37] Chikhladze S , Lederer A‐K , Kousoulas L , et al. Adjuvant chemotherapy after surgery for pancreatic ductal adenocarcinoma: retrospective real‐life data. World J Surg Oncol. 2019;17(1):185. 31706323 10.1186/s12957-019-1732-3 PMC6842534

[38] Neoptolemos JP , Palmer DH , Ghaneh P , et al. Comparison of adjuvant gemcitabine and capecitabine with gemcitabine monotherapy in patients with resected pancreatic cancer (ESPAC‐4): a multicentre, open‐label, randomised, phase 3 trial. Lancet. 2017;389(10073):1011‐1024. 28129987 10.1016/S0140-6736(16)32409-6

[39] Tzeng C‐WD , Cao HST , Lee JE , et al. Treatment sequencing for resectable pancreatic cancer: influence of early metastases and surgical complications on multimodality therapy completion and survival. J Gastrointest Surg. 2014;18(1):16‐25. 24241967 10.1007/s11605-013-2412-1

[40] O'Reilly EM , Ferrone C . Neoadjuvant or adjuvant therapy for resectable or borderline resectable pancreatic cancer: which is preferred? J Clin Oncol. 2020;38(16):1757‐1759. 32119598 10.1200/JCO.19.03318 PMC7255981

[41] Valsangkar NPBD , Michaelson JS , Ferrone CR , Wargo JA , Lillemoe KD . N0/N1, PNL, or LNR? The effect of lymph node number on accurate survival prediction in pancreatic ductal adenocarcinoma. J Gastrointest Surg. 2013;17(12):257‐266. 23229885 10.1007/s11605-012-1974-7 PMC3806050

[42] Huebner MKM , Reid‐Lombardo KM , Que F , et al. Number of lymph nodes evaluated: prognostic value in pancreatic adenocarcinoma. J Gastrointest Surg. 2012;16(15):920‐926. 22421988 10.1007/s11605-012-1853-2

[43] Slidell MB , Chang DC , Cameron JL , et al. Impact of total lymph node count and lymph node ratio on staging and survival after pancreatectomy for pancreatic adenocarcinoma: a large. Population‐Based Anal. 2007;15(1):165. 10.1245/s10434-007-9587-1 17896141

[44] Pawlik TM , Gleisner AL , Cameron JL , et al. Prognostic relevance of lymph node ratio following pancreaticoduodenectomy for pancreatic cancer. Surgery. 2007;141(5):610‐618. 17462460 10.1016/j.surg.2006.12.013

[45] Regine WF , Winter KA , Abrams RA , et al. Fluorouracil vs gemcitabine chemotherapy before and after fluorouracil‐based chemoradiation following resection of pancreatic adenocarcinoma: a randomized controlled trial. JAMA. 2008;299(9):1019‐1026. 18319412 10.1001/jama.299.9.1019

[46] Jones RP , Psarelli E‐E , Jackson R , et al. Patterns of recurrence after resection of pancreatic ductal adenocarcinoma: a secondary analysis of the ESPAC‐4 randomized adjuvant chemotherapy trial. JAMA Surg. 2019;154(11):1038‐1048. 31483448 10.1001/jamasurg.2019.3337 PMC6727687

[47] Tempero MA , Reni M , Riess H , et al. APACT: phase III, multicenter, international, open‐label, randomized trial of adjuvant nab‐paclitaxel plus gemcitabine (nab‐P/G) vs gemcitabine (G) for surgically resected pancreatic adenocarcinoma. J Clin Oncol. 2019;37(15_suppl):4000.

[48] Rochefort MM , Ankeny JS , Kadera BE , et al. Impact of tumor grade on pancreatic cancer prognosis: validation of a novel TNMG staging system. Ann Surg Oncol. 2013;20(13):4322‐4329. 23943022 10.1245/s10434-013-3159-3

[49] Wasif N , Ko CY , Farrell J , et al. Impact of tumor grade on prognosis in pancreatic cancer: should we include grade in AJCC staging? Ann Surg Oncol. 2010;17(9):2312‐2320. 20422460 10.1245/s10434-010-1071-7 PMC2924500

[50] Brennan MF , Kattan MW , Klimstra D , Conlon K . Prognostic nomogram for patients undergoing resection for adenocarcinoma of the pancreas. Ann Surg. 2004;240(2):293‐298. 15273554 10.1097/01.sla.0000133125.85489.07 PMC1356406

[51] Lau MK , Davila JA , Shaib YH . Incidence and survival of pancreatic head and body and tail cancers: a population‐based study in the United States. Pancreas. 2010;39(4):458‐462. 19924019 10.1097/MPA.0b013e3181bd6489

[52] Watanabe I , Sasaki S , Konishi M , et al. Onset symptoms and tumor locations as prognostic factors of pancreatic cancer. Pancreas. 2004;28(2):160‐165. 15028948 10.1097/00006676-200403000-00007

[53] Artinyan A , Soriano PA , Prendergast C , Low T , Ellenhorn JDI , Kim J . The anatomic location of pancreatic cancer is a prognostic factor for survival. HPB. 2008;10(5):371‐376. 18982154 10.1080/13651820802291233 PMC2575681

[54] Tomasello G , Ghidini M , Costanzo A , et al. Outcome of head compared to body and tail pancreatic cancer: a systematic review and meta‐analysis of 93 studies. J Gastrointest Oncol. 2019;10(2):259‐269. 31032093 10.21037/jgo.2018.12.08 PMC6465486

[55] Dreyer SB , Jamieson NB , Upstill‐Goddard R , et al. Defining the molecular pathology of pancreatic body and tail adenocarcinoma. Br J Surg. 2018;105(2):e183‐e191. 29341146 10.1002/bjs.10772 PMC5817249

[56] Bapat AA , Hostetter G , Von Hoff DD , Han H . Perineural invasion and associated pain in pancreatic cancer. Nat Rev Cancer. 2011;11(10):695‐707. 21941281 10.1038/nrc3131

[57] Ethun CG , Kooby DA . The importance of surgical margins in pancreatic cancer. J Surg Oncol. 2016;113(3):283‐288. 26603829 10.1002/jso.24092

[58] Ghaneh P , Kleeff J , Halloran CM , et al. The impact of positive resection margins on survival and recurrence following resection and adjuvant chemotherapy for pancreatic ductal adenocarcinoma. Ann Surg. 2019;269(3):520‐529. 29068800 10.1097/SLA.0000000000002557

[59] Tummers WS , Groen JV , Sibinga Mulder BG , et al. Impact of resection margin status on recurrence and survival in pancreatic cancer surgery. Br J Surg. 2019;106(8):1055‐1065. 30883699 10.1002/bjs.11115 PMC6617755

[60] Raut CP , Tseng JF , Sun CC , et al. Impact of resection status on pattern of failure and survival after pancreaticoduodenectomy for pancreatic adenocarcinoma. Ann Surg. 2007;246(1):52‐60. 17592291 10.1097/01.sla.0000259391.84304.2b PMC1899216

[61] Kato K , Yamada S , Sugimoto H , et al. Prognostic factors for survival after extended pancreatectomy for pancreatic head cancer: influence of resection margin status on survival. Pancreas. 2009;38(6):605‐612. 19629002 10.1097/MPA.0b013e3181a4891d

[62] Jamieson NB , Foulis AK , Oien KA , et al. Positive mobilization margins alone do not influence survival following pancreatico‐duodenectomy for pancreatic ductal adenocarcinoma. Ann Surg. 2010;251(6):1003‐1010. 20485150 10.1097/SLA.0b013e3181d77369

[63] Versteijne E , Suker M , Groothuis K , et al. Preoperative chemoradiotherapy versus immediate surgery for resectable and borderline resectable pancreatic cancer: results of the dutch randomized Phase III PREOPANC trial. J Clin Oncol. 2020;38(16):1763‐1773. 10.1200/jco.19.02274. 32105518 PMC8265386

[64] Unno M , Motoi F , Matsuyama Y , et al. Randomized phase II/III trial of neoadjuvant chemotherapy with gemcitabine and S‐1 versus upfront surgery for resectable pancreatic cancer (Prep‐02/JSAP‐05). J Clin Oncol. 2019;37(4_suppl):189‐189. 10.1200/jco.2019.37.4_suppl.189. 30608598

[65] Sohal D , Duong MT , Ahmad SA , et al. SWOG S1505: Results of perioperative chemotherapy (peri‐op CTx) with mfolfirinox versus gemcitabine/nab‐paclitaxel (Gem/nabP) for resectable pancreatic ductal adenocarcinoma (PDA). J Clin Oncol. 2020;38(15_suppl):4504‐4504. 10.1200/jco.2020.38.15_suppl.4504.

