# Supplementary material for: Resected pancreatic adenocarcinoma: An Asian institution's experience
Source: Cancer Rep (Hoboken). 2021 May 3;4(5):e1393. doi: 10.1002/cnr2.1393 (PMC8551988; doi:10.1002/cnr2.1393)
Supplement: Supplementary file 1 — Figure S1. Recurrence pattern. [file CNR2-4-e1393-s001.docx]

**Figure S1. Recurrence Pattern**
